# Excellent survivorship and functional outcomes following lateral unicompartmental knee arthroplasty in patients aged 55 years or younger

**DOI:** 10.1002/jeo2.70935

**Published:** 2026-10-03

**Authors:** Solongo Abdulai, Conradin Schweizer, Valentin Rühle, Tatjana Krug, Raffaela Morgenbesser, Joachim Herre, Peter R. Aldinger, Christian Merle, Wenzel Waldstein

**Affiliations:** ^1^ Orthopädische Klinik Paulinenhilfe Diakonie Klinikum Stuttgart Stuttgart Germany; ^2^ Department of Orthopedics and Traumatology Paracelsus Medical University of Salzburg Salzburg Austria

**Keywords:** lateral compartment osteoarthritis, lateral unicompartmental knee arthroplasty, survivorship, young patients

## Abstract

**Purpose:**

Fixed‐bearing lateral unicompartmental knee arthroplasty (UKA) is a bone‐ and ligament‐preserving treatment option for isolated lateral‐compartment osteoarthritis (OA), but evidence in younger patients remains limited. The primary purpose of this study was to assess lateral component revision‐free survival and reoperation‐free survival following primary fixed‐bearing lateral UKA in patients aged 55 years or younger. Secondary objectives were to evaluate functional and radiological outcomes.

**Methods:**

This retrospective case series was derived from a prospectively maintained institutional database. A consecutive series of 84 primary fixed‐bearing lateral UKAs in 80 patients aged ≤55 years, performed between January 2016 and December 2021 with a minimum follow‐up of 2 years was identified (mean follow‐up, 5.3 ± 1.9 years [range, 2.2–8.6 years]; mean age, 51 years [range, 37–55 years]). Kaplan‐Meier analysis estimated 5‐year survival for lateral component revision or reoperation. Lateral component revision was defined as the removal or exchange of any fixed implant component (femoral or tibial). Reoperation was defined as any subsequent surgical procedure on the operated knee. Functional outcomes were evaluated using the Oxford Knee Score (OKS) and University of California, Los Angeles Activity Score. Radiographic assessment included measurement of the hip‐knee‐ankle angle (HKAA).

**Results:**

Lateral component revision‐free survival was 100%, while cumulative reoperation‐free survivorship was 92.5% (95% CI: 86.8–98.2) at 5 years. Seven reoperations were observed: 4 additional medial UKAs, 2 arthroscopic procedures and 1 successful debridement, antibiotics and implant retention (DAIR). Mean HKAA changed from 4.8° ± 4.7° valgus preoperatively to 2.0° ± 2.8° valgus postoperatively. Postoperative HKAA demonstrated higher residual valgus in knees without medial‐compartment OA progression or osteonecrosis (ON) compared to four knees with medial‐compartment OA or ON (2.2° valgus vs. 1.5° varus, *p* = 0.012). The mean postoperative OKS was 43.8 ± 5.7 and the UCLA Activity Score was 6.5 ± 1.2.

**Conclusions:**

Fixed‐bearing lateral UKA in younger patients was associated with 100% lateral component revision‐free survival and high functional scores at a mean follow‐up of 5 years. No implant‐related failures, such as aseptic loosening, were observed in this active cohort, although seven knees underwent reoperation, including four additional medial UKAs. Progression of OA in the medial compartment was the most common reason for reoperation. The observed association between postoperative varus alignment and medial‐compartment OA progression or ON is limited by the small number of events and requires further investigation.

**Level of Evidence:**

Level IV.

## Graphical abstract

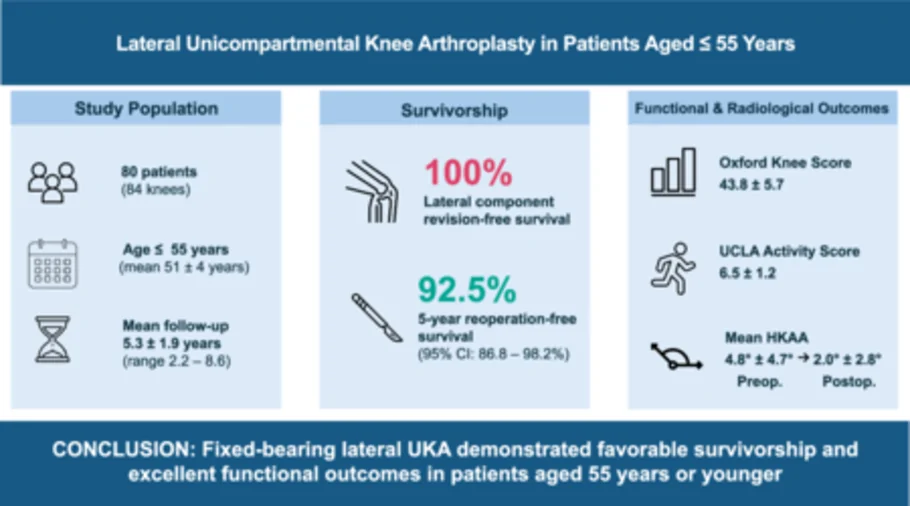

**Abbreviations**
ACL: anterior cruciate ligament
AP: anteroposterior
BMI: body mass index
CI: confidence interval
DAIR: debridement, antibiotics and implant retention
DLFA: distal lateral femoral angle
HKAA: hip‐knee‐ankle angle
IRB: institutional review board
JLCA: joint line convergence angle
JLO: joint line obliquity
MPTA: medial proximal tibial angle
MRI: magnetic resonance imaging
OA: osteoarthritis
OKS: Oxford Knee Score
ON: osteonecrosis
ORIF: open reduction and internal fixation
PLOA: primary lateral osteoarthritis
PROMs: patient‐reported outcome measures
TKA: total knee arthroplasty
UCLA: University of California, Los Angeles Activity Score
UKA: unicompartmental knee arthroplasty

## INTRODUCTION

Fixed‐bearing lateral unicompartmental knee arthroplasty (UKA) is an established treatment option for isolated lateral‐compartment osteoarthritis (OA) and represents a bone‐ and ligament‐preserving alternative to total knee arthroplasty (TKA) in appropriately selected patients [1, 31]. Compared to TKA, UKA in general has been associated with better patient‐reported outcome measures (PROMs) [11], faster recovery, reduced morbidity and mortality [11, 15] and greater cost‐effectiveness [4, 27]. Recent studies have reported high survivorship, low reoperation rates and favourable functional outcomes for contemporary fixed‐bearing lateral UKA designs [2, 21, 26, 29]. Despite these reported advantages, fixed‐bearing lateral UKA remains an infrequently performed procedure. This has primarily been attributed to limited evidence for indication criteria and higher surgical complexity [1, 3].

Previous knee trauma or lateral meniscectomy predispose to early lateral‐compartment OA [6, 17]. Consequently, fixed‐bearing lateral UKA appears to be a promising alternative to TKA in the distinct subpopulation of younger patients with high functional demands. A recent study from the authors' institution reported excellent mid‐term outcomes of fixed‐bearing lateral UKA in a large consecutive single‐centre cohort across all age groups [29]. However, evidence regarding the outcomes of fixed‐bearing lateral UKA in younger patients remains limited [9, 10, 23, 24]. To date, no previous study has specifically evaluated the outcome of fixed‐bearing lateral UKA in patients aged 55 years or younger.

Therefore, the primary purpose of the present study was to evaluate lateral component revision‐free survival and reoperation‐free survival following primary fixed‐bearing lateral UKA in patients aged 55 years or younger. Secondary objectives were to assess PROMs and radiological outcomes. It was hypothesised that fixed‐bearing lateral UKA in patients aged 55 years or younger would be associated with high midterm component revision‐free survival and favourable postoperative PROMs.

## METHODS

The present cohort represents an age‐specific retrospective case series of a previously published consecutive single‐centre cohort based on a prospectively maintained institutional database [29] (Figure 1). While the previous study reported the overall outcomes of fixed‐bearing lateral UKA across all age groups, the present study represents a subsequent, novel subgroup analysis focusing on the clinically distinct subpopulation of patients aged 55 years or younger. Patients were eligible for inclusion if they had undergone primary fixed‐bearing lateral UKA between January 2016 and December 2021, were aged 55 years or younger at the time of surgery, and had a minimum follow‐up of 2 years. Patients who declined or withdrew informed consent were excluded. Institutional review board approval was obtained (IRB_003/2023), and written informed consent was provided by all participants.

**Figure 1:**
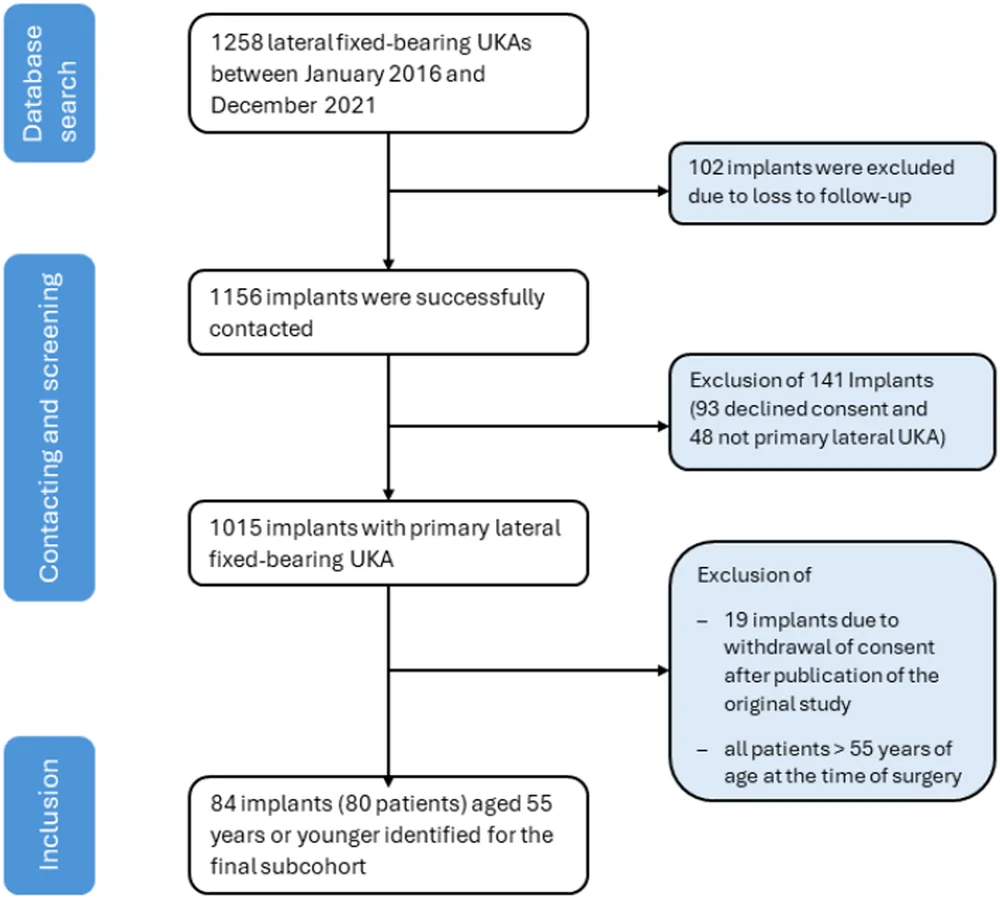
Flowchart of patient selection for the final study cohort. UKA, unicompartmental knee arthroplasty.

### Patient selection and preoperative imaging

All patients were evaluated following a standardised radiographic protocol including anteroposterior (AP) and lateral knee radiographs, patellar skyline views and varus/valgus stress radiographs. The established indication criteria for medial mobile‐bearing UKA [8] were adapted, and overall limb alignment was not considered a determining factor for the indication of fixed‐bearing lateral UKA. Fixed‐bearing lateral UKA was offered to all patients who demonstrated full‐thickness lateral‐compartment cartilage loss on valgus stress view, a fully correctable valgus deformity, an intact medial compartment on varus stress views and an extension deficit of less than 10°. Functional anterior cruciate ligament integrity was assessed clinically during preoperative examination and reassessed intraoperatively. In addition to clinical examination, collateral ligament integrity was evaluated using standardised varus and valgus stress radiographs. Medial knee pain in the presence of valgus alignment, likely attributable to medial collateral ligament stretching, was not considered a contraindication to lateral UKA. Patellofemoral joint status was not considered relevant unless severe and symptomatic medial patellofemoral OA associated with bone loss, grooving or patellar maltracking was present. In selected cases, Rosenberg radiographs (45° flexion posteroanterior weight‐bearing radiographs) and/or magnetic resonance imaging (MRI) were obtained to further assess suitability for fixed‐bearing lateral UKA. Rheumatoid arthritis and a history of previous periarticular osteotomy were considered relative contraindications. In contrast, body weight [32], activity level [22] and medial‐compartment chondrocalcinosis [14, 18] were not regarded as contraindications. AP standing hip‐to‐ankle radiographs were routinely obtained prior to surgery.

### Surgical technique and postoperative care

The cemented Oxford® Fixed Lateral Partial Knee (Zimmer‐Biomet) was used in all cases. The leg was positioned at approximately 110° of knee flexion with the hip flexed to approximately 30° using a thigh support and an attached thigh tourniquet, which was inflated in all cases. Fixed‐bearing lateral UKA was implanted using a minimally invasive lateral parapatellar approach with the vertical incision performed through the ligamentum patellae to achieve approximately 20°–30° of internal tibial rotation. Ligament balancing aimed to achieve stability in full extension while preserving physiological laxity in any degree of flexion. The target was to reconstruct the preoperative constitutional leg alignment while avoiding any releases. Preoperative stress radiographs were taken into consideration when balancing the knee. The surgical technique remained unchanged throughout the study period. A total of 99% of procedures (83/84) were performed by five high‐volume surgeons (>100 UKAs/year). Early postoperative mobilisation and full weight‐bearing as tolerated were encouraged. During the initial rehabilitation phase, patients were instructed to avoid forced knee flexion and leg press exercises to protect the lateral joint capsule. Before discharge, true AP and lateral knee radiographs were obtained under fluoroscopic guidance.

### Implant survival, clinical and radiographic evaluation

A standardised patient telephone interview was conducted between December 2023 and February 2025 to obtain information on implant survival, any reoperation and postoperative PROMs. The date of the telephone interview was considered the date of final follow‐up. Postoperative PROMs included the Oxford Knee Score (OKS) and the University of California, Los Angeles (UCLA) Activity Score, which are both established instruments for evaluating function and activity following knee arthroplasty [19, 20]. Baseline OKS and UCLA Activity Score values were available for 15 of 84 knees (18%) of the cohort. Postoperative PROMs were available in all cases.

Preoperatively, the hip‐knee‐ankle angle (HKAA), medial proximal tibial angle (MPTA) and distal lateral femur angle (DLFA) were measured on AP standing hip‐to‐ankle radiographs as previously described [25, 28]. The joint line convergence angle (JLCA) was measured on preoperative varus stress views and was defined as the angle between a best‐fit line along the surface of the tibial plateau and a line connecting the most distal points of the medial and lateral femoral condyles. All postoperative radiographs were obtained within the first three postoperative days. Postoperative HKAA, joint line obliquity (JLO) and the coronal tibial component angle were measured on reconstructed hip‐to‐ankle radiographs using a validated and previously reported technique [25]. Preoperative MPTA and postoperative JLO were considered comparable. Radiographic measurements were performed by independent observers who were blinded to the clinical outcomes and reoperation status. Pre and postoperative radiographic measurements were performed using JiveX (VISUS Health IT GmbH) and OsiriX (Pixmeo). Measurement reliability for postoperative HKAA assessment was excellent: the intraobserver intraclass correlation coefficient was 0.998 (95% CI: 0.994–0.999), and the interobserver intraclass correlation coefficient was 0.981 (95% CI: 0.952–0.992), respectively.

### Statistics

Continuous variables and scores were expressed as means with standard deviation, while categorical variables were presented as absolute numbers and percentages. Kaplan–Meier survival analysis was conducted for the endpoints (1) lateral component revision‐free survival and (2) any reoperation. Given the limited number of patients at risk beyond 5 years, survivorship estimates are reported at 5 years, while the Kaplan–Meier curve is displayed over the complete available follow‐up period. Lateral component revision was defined as removal or exchange of the tibial and/or femoral component. Reoperation was defined as any subsequent surgical intervention on the operated knee, including implantation of an additional UKA for medial‐compartment OA progression or osteonecrosis (ON). The normality of data was assessed using the Shapiro–Wilk test. Depending on data distribution, either a *t*‐test or Mann–Whitney *U*‐test was applied. For paired comparison, the Wilcoxon signed‐rank test or paired *t*‐test was performed. *P*‐values of < 0.05 were considered statistically significant. All statistical analyses were performed using IBM SPSS Statistics, version 30 (IBM Corp.).

## RESULTS

Of the 1258 fixed‐bearing lateral UKAs performed during the study period, 107 knees (103 patients) were implanted in patients aged 55 years or younger at the time of surgery. Of these, 88 knees (84 patients) were successfully contacted for follow‐up, corresponding to a follow‐up rate of 82% (88/107). A total of 19 knees (19 patients) were lost to follow‐up. Four knees were further excluded as they did not meet the study eligibility criteria. The final analysis included 84 knees in 80 patients aged 55 years or younger at the time of surgery (Figure 1, Table 1).

**Table 1:** Baseline demographic and clinical characteristics of patients undergoing primary lateral unicompartmental knee arthroplasty.

Variable	Value
Patients/knees, *n*	80/84
Bilateral cases, *n* (%)	4 (5)
Left/right, *n* (%)	36 (43)/48 (57)
Age, years (range)	51 ± 4.2 (37–55)
BMI, kg/m^2^	28.9 ± 5.6 (18.7–43.9)
Female/male, *n* (%)	55 (66)/29 (34)
Follow‐up, years (range)	5.3 ± 1.9 (2.2–8.6)
Etiology, *n* (%)	
Lateral‐compartment OA	78 (93)
Lateral osteonecrosis	1 (1)
Posttraumatic OA	5 (6)
Preoperative alignment, *n* (%)	
Valgus/varus	74 (88)/10 (12)
Mean HKAA, ° (range)	4.8 valgus ± 4.7 (17.5 valgus–5.3 varus)
Previous knee surgery, *n* (%)	61 (73)
Lateral meniscus surgery	40 (66)
Arthroscopic procedure, not further specified	13 (21)
ORIF of a lateral tibial plateau fracture	4 (7)
Anterior cruciate ligament reconstruction	2 (3)
Medial meniscus surgery	1 (1.5)
Knee surgery not further specified	1 (1.5)

*Note*: Data are presented as mean ± standard deviation (range) or *n* (%).

Abbreviations: BMI, body mass index; HKAA, hip–knee–ankle angle; OA, osteoarthritis, ORIF, open reduction and internal fixation.

At a mean follow‐up of 5.3 years (range: 2.2–8.6), no lateral component revision was observed, resulting in a lateral component revision‐free survival of 100%. The cumulative 5‐year reoperation‐free survivorship was 92.5% (95% CI: 86.8–98.2) (Figure 2). The latest reoperation occurred at 5.8 years of follow‐up (number at risk: 30). In the study cohort, previous surgery was performed in 73% (61/84) of the knees in 59 patients (Table 1). The average time between previous surgery and fixed‐bearing lateral UKA was 11.9 ± 10.9 years.

**Figure 2:**
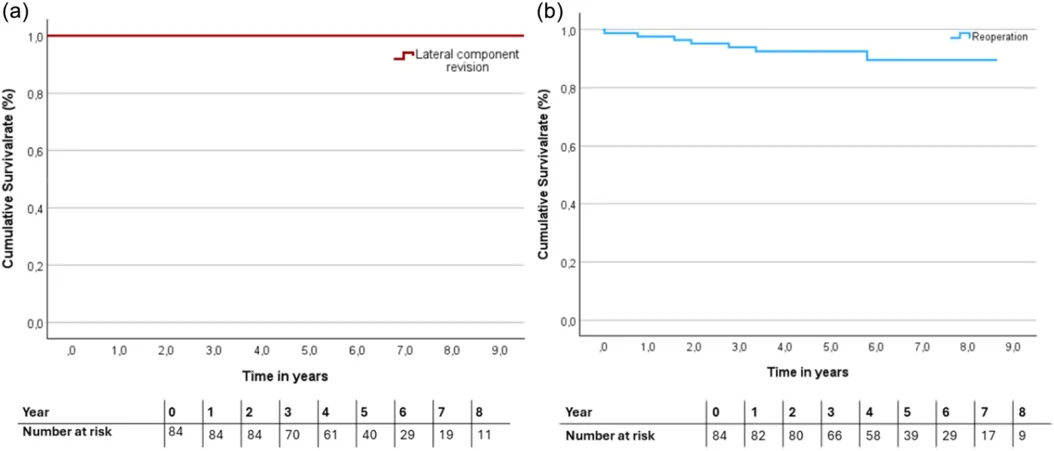
Kaplan–Meier curves showing lateral component revision‐free and reoperation‐free survival with corresponding number at risk tables. The cumulative lateral component revision‐free survival was 100% at 5 years (a) and the cumulative 5‐year reoperation‐free survival was 92.5% (95% CI: 86.8–98.2) (b).

Overall, seven reoperations were observed during the study period: one arthroscopic procedure for a degenerative medial meniscal lesion, one arthroscopic synovectomy and patella osteophyte removal, one debridement, antibiotics and implant retention (DAIR) procedure for early postoperative infection caused by *β‐hemolytic streptococci* and *Staphylococcus aureus* 15 days after index surgery. Four additional medial UKAs were performed, including three for medial‐compartment OA progression and one for ON of the medial femoral condyle (Table 2). Implantation of an additional medial UKA represented the most common reoperation (57%, 4/7), with medial‐compartment OA progression being the most frequent reason for reoperation (43%, 3/7). Among the four patients who developed medial‐compartment OA progression or ON, one was overcorrected from 7.3° valgus to 2.1° varus. In the second patient, a mild preoperative valgus deformity of 2° was corrected to 0.9° valgus postoperatively. The third patient already had a preoperative varus alignment of 0.6°, which was kept at 0.5° varus postoperatively. The fourth patient with a preoperative varus of 1.1°, which was further increased to 4.2° varus developed an ON of the medial femoral condyle, which required an additional medial UKA.

**Table 2:** Characteristics of the seven reoperated patients following fixed‐bearing lateral unicompartmental knee arthroplasty (UKA).

Reoperation	Age (years)	Sex	Previous surgery	Index diagnosis	Preoperative HKAA	Postoperative HKAA	Time to reoperation (months)	Indication for reoperation	Performed procedure
1	47	m	Lateral meniscus surgery	PLOA	2.9° varus	3.1° varus	0.5	Early infection	DAIR
2	49	f	Lateral meniscus surgery	PLOA	7.8° valgus	4.1° valgus	69.5	Medial meniscal lesion	Arthroscopy
3	51	f	Lateral meniscus surgery	PLOA	2.4° valgus	1.9° valgus	18.8	Synovitis and patellar osteophyte	Arthroscopy
4	49	f	Lateral meniscus surgery	PLOA	7.3° valgus	2.1° varus	33.1	OA progression	Additional medial UKA
5	52	m	Lateral meniscus surgery	PLOA	2.0° valgus	0.9° valgus	40.3	OA progression	Additional medial UKA
6	42	f	Lateral meniscus surgery	PLOA	0.6° varus	0.5° varus	9.2	OA progression	Additional medial UKA
7	55	m	Lateral meniscus surgery	PLOA	1.1° varus	4.2° varus	23.3	Osteonecrosis	Additional medial UKA

Abbreviations: DAIR, debridement, antibiotics and implant retention; f, female; HKAA, hip–knee–ankle angle; m, male; OA, osteoarthritis; PLOA, primary lateral osteoarthritis; UKA, unicompartmental knee arthroplasty.

The overall mean postoperative OKS was 43.8 ± 5.7 and the mean postoperative UCLA Activity Score was 6.5 ± 1.2 (*n* = 84). Among the 15 knees with available paired pre and postoperative PROMs, the mean OKS improved from 24.3 ± 9.2 preoperatively to 44.1 ± 5.4 postoperatively (*p* < 0.001). Similarly, the mean UCLA Activity Score increased from 5.8 ± 2.5 to 6.7 ± 1.4, although this change did not reach statistical significance (*p* = 0.119). Fixed‐bearing lateral UKA corrected the mean preoperative HKAA of 4.8° ± 4.7° valgus to 2.0° ± 2.8° valgus postoperatively (*p* < 0.001). Patients without medial‐compartment OA progression or ON had a mean HKAA of 5.0° ± 4.7° valgus preoperatively and 2.2° ± 1.7° valgus postoperatively. In contrast, patients who developed medial‐compartment OA progression or ON had a mean preoperative HKAA of 1.8° ± 4.9° valgus and a postoperative HKAA of 1.5° ± 2.2° varus. Descriptively, knees with subsequent medial‐compartment OA progression or ON, therefore, showed a postoperative alignment which was more in varus. In an exploratory comparison, postoperative HKAA differed between knees without and with medial‐compartment OA progression or ON (2.2° ± 1.7° valgus vs. 1.5° ± 2.2° varus, *p* = 0.012) (Figure 3). Overall, 14 out of 84 (17%) knees had mild postoperative varus alignment. Among knees with preoperative valgus alignment, 7 of 84 (8%) showed postoperative varus, including one knee that subsequently required reoperation for medial‐compartment OA progression. Ten knees (12%) presented with preoperative varus alignment, of which seven remained in varus postoperatively. The characteristics of these patients are summarised in Table 3. Two of these ten knees (20%) subsequently developed medial‐compartment OA or ON, compared with two of the remaining 74 knees (3%) without preoperative varus alignment.

**Figure 3:**
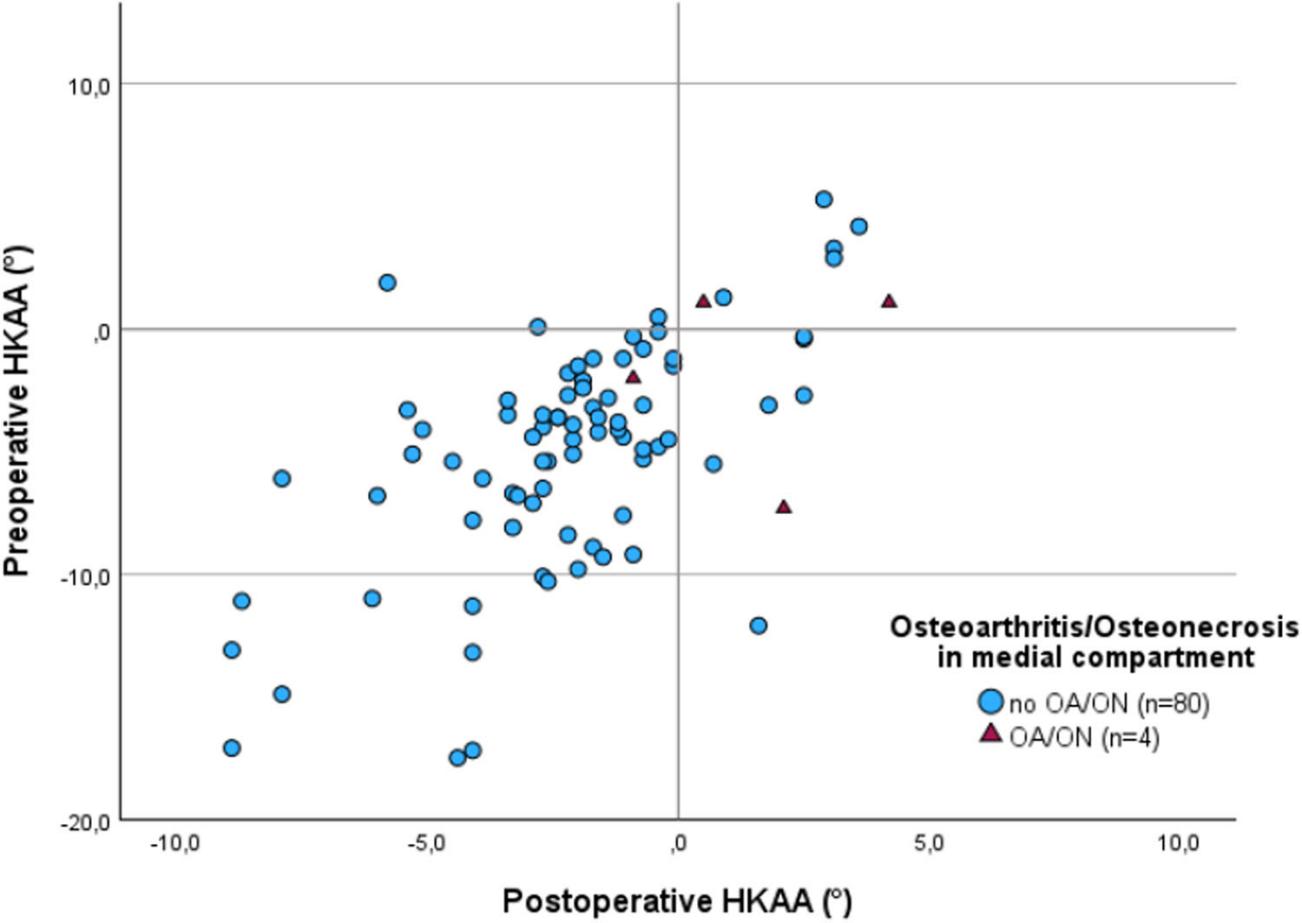
Scatter plot showing the relationship between preoperative and postoperative hip‐knee‐ankle angle (HKAA). Blue points show no OA/ON in the medial compartment while red triangles illustrate medial OA/ON. Negative values represent valgus alignment and positive values varus alignment. Three of four cases with medial OA/ON had a postoperative varus alignment. OA, osteoarthritis; ON, osteonecrosis.

**Table 3:** Patient characteristics and outcomes of the 10 knees with preoperative varus alignment.

Nr.	Age (years)	Sex	BMI (kg/m^2^)	Follow‐up (years)	Previous lateral procedure	Preop. HKAA (varus)	Postop. HKAA	Medial OA/ON	Further arthroplasty
1	43	m	29.9	2.9	Lateral meniscus surgery	1.9°	5.8° valgus	No	No
2	37	f	26.1	2.7	ORIF of a lateral tibial plateau fracture	1.3°	0.9° varus	No	No
3	42	f	41.6	4.0	Lateral meniscus surgery	0.6°	0.5° varus	Yes	Yes
4	52	m	30.7	4.8	Lateral meniscus surgery	0.5°	0.4° valgus	No	No
5	47	m	25.5	4.7	Lateral meniscus surgery	3.3°	3.1° varus	No	No
6	47	m	25.5	4.7	Lateral meniscus surgery	5.3°	2.9° varus	No	No
7	55	m	35.5	3.1	Lateral meniscus surgery	1.1°	4.2° varus	Yes	Yes
8	52	f	42.2	5.6	Lateral meniscus surgery	4.2°	3.6° varus	No	No
9	53	m	27.2	7.2	ORIF of a lateral tibial plateau fracture	0.1°	2.8° valgus	No	No
10	47	m	26.0	8.3	Lateral meniscus surgery	2.9°	3.1° varus	No	No

Abbreviations: BMI, body‐mass‐index; f, female; HKAA, hip–knee–ankle angle; m, male; OA, osteoarthritis; ON, osteonecrosis; ORIF, open reduction and internal fixation; postop., postoperative; preop., preoperative.

The mean preoperative MPTA was 89.5° ± 2.8° and the mean preoperative DLFA was 86.1° ± 2.4°. Postoperatively, the tibial component angle was 90.5° ± 2.6°, while the JLO measured 87.2° ± 2.1°. Overall, the postoperative JLO was more in varus (mean 2.3° ± 2.5°) compared to the preoperative MPTA (*p* < 0.001).

## DISCUSSION

The most important finding of the present study was that fixed‐bearing lateral UKA in patients aged 55 years or younger was associated with 100% lateral component revision‐free survival and favourable functional outcomes at a mid‐term follow‐up of 5 years. No implant‐related failures, such as aseptic loosening were observed in this younger cohort. Seven knees underwent reoperation, including four additional medial UKAs for medial‐compartment OA progression or ON, with medial‐compartment OA progression representing the most common indication for reoperation (43%, 3/7).

There is limited evidence regarding the mid‐to‐long‐term performance of fixed‐bearing lateral UKA in younger patients (Table 4). Sangaletti et al. analysed implant survivorship of 40 fixed‐bearing lateral UKAs in patients at a mean age of 58 years at the time of surgery [24]. Reoperation‐free survival in their study was 93% at 10 years. Hartman et al. described the survival rates of fixed‐ and mobile‐bearing lateral UKA in 49 patients at a mean age of 55 years [10]. Hartman observed a lower 10‐year survivorship (86%) for the endpoint conversion to TKA, which was attributed to the higher revision rates of mobile‐bearing implants. Several studies have reported on the survivorship of medial UKA in younger patients. Previous studies in similarly younger cohorts with a mean age of approximately 50 years reported 10‐year survivorship rates of 90.4% and 91.7% [5, 30]. However, a comparison between medial and lateral UKA is difficult due to the distinct anatomical and biomechanical differences of the medial and lateral compartment. The present study evaluated the largest and one of the youngest cohorts reported to date, comprising 80 patients (84 knees) who underwent fixed‐bearing lateral UKA at a mean age of 51 years (range, 37–55 years). Lateral component revision‐free survival was 100%, and reoperation‐free survivorship was 92.5% at mid‐term follow‐up. Despite the high activity level of younger patients, no aseptic implant loosening was observed. Overall, seven knees required additional surgery during the study period. Apart from two arthroscopic procedures and one successful DAIR, four cases of medial‐compartment OA progression or ON were observed, all of which resulted in the addition of a medial UKA. While the four additional medial UKAs did not meet the definition of lateral component revision, they represent clinically relevant secondary arthroplasty procedures and should be considered accordingly when interpreting the 100% lateral component revision‐free survival.

**Table 4:** Summary of previous studies reporting outcomes of lateral unicompartmental knee arthroplasty in younger patients.

Study	Patients (*n*)	Age (years)	Implant	Follow‐up (years)	Outcome
Sangaletti et al. [24]	40	58 ± 9	Fixed‐bearing	11.1 ± 1.9	93% at 10 years for any reoperation
Hartman et al. [10]	49	55 (45–82)	Fixed‐/mobile‐bearing	8.8 (1.7–12.2)	86% at 10 years for conversion to TKA
Plancher et al. [23]	18	51 ± 5	Fixed‐bearing	11.0 ± 3.0	100% at 10 years for conversion to TKA
Hariri et al. [9]	37	53 ± 3	Fixed‐bearing	3.1 ± 1.5	100% for implant revision

*Note*: Age and follow‐up are reported as mean ± standard deviation or mean (range), according to the data available in the respective studies.

Abbreviation: TKA, total knee arthroplasty.

The majority of knees had previously undergone arthroscopic treatment of the lateral meniscus, which is a known risk factor for early‐onset OA in the lateral compartment [17]. Compared to knees indicated for fixed‐bearing lateral UKA in older patients with preoperative limb alignment of 7°–11° valgus [16, 21, 29], patients in this younger cohort presented with less severe valgus of 5°. These knees with a history of previous surgery and only mild native valgus or varus alignment appear to represent a distinct clinical entity. Additionally, these knees are at higher risk for postoperative varus overcorrection. In 8% of cases in this study (7/84), correction from preoperative valgus to postoperative varus alignment was observed, which is a previously described risk factor for OA progression and ON in the medial compartment [26, 29]. In this context, the present study showed that the four knees with medial‐compartment OA progression or ON had a mean postoperative HKAA of 1.5° varus; three demonstrated postoperative varus alignment, while one was corrected close to neutral (Figure 3).

Balancing a fixed‐bearing lateral UKA with stability in full extension and laxity in flexion can be challenging. Khamaisy et al. showed that fixed‐bearing lateral UKA carries a significantly greater risk of overcorrection than medial UKA (11% vs. 4%, *p* = 0.0001) [13]. Although the number of events was limited, the findings of this study are consistent with recently published literature suggesting a potential association between postoperative near‐neutral or varus alignment and medial‐compartment OA progression or ON [16, 29]. The question of ‘classic’ and extended indications for fixed‐bearing lateral UKA is still unanswered as well‐defined and consensus‐based indications for fixed‐bearing lateral UKA have not yet been established [12]. In this cohort, fixed‐bearing lateral UKA was also performed in knees with isolated lateral‐compartment OA and mild preoperative varus leg alignment. To the best of the authors' knowledge, this has not previously been described in the literature. All of these 10 cases (12%) had a previous lateral meniscus resection or surgical management of a lateral tibia plateau fracture. Two of these knees with preoperative varus later developed medial‐compartment OA/ON. Although this small sample size precludes definitive conclusions, these findings suggest that fixed‐bearing lateral UKA may be a reasonable treatment option in carefully selected patients with mild preoperative varus alignment. However, these patients should be carefully counselled about the potentially higher risk of medial‐compartment OA progression, and particular attention should be paid to avoid postoperative overcorrection.

Garner et al. reported more physiological gait characteristics following fixed‐bearing lateral UKA compared with TKA, including faster walking speeds and more physiological vertical ground reaction forces [7]. In the present study, postoperative functional outcomes were favourable, with a mean postoperative OKS of 44 and UCLA Activity Score of seven. However, preoperative PROMs were available for only a limited subset of knees, restricting the assessment of longitudinal functional improvement.

This study has several limitations. Its retrospective design, the absence of a comparator group and the high‐volume single‐centre setting may introduce bias and limit generalisability. The present cohort represents an age‐specific subgroup of a previously published institutional cohort [29], and the follow‐up rate of 82% should be considered when interpreting the present findings. Preoperative PROMs were available for only 18% (15/84) of knees, limiting longitudinal functional assessment, while the telephone‐based assessment of postoperative PROMs represents an additional limitation. Furthermore, medial‐compartment OA progression was captured through reoperations, as systematic longitudinal radiographic follow‐up was not available. Additionally, postoperative alignment was assessed using hip‐to‐ankle radiographs reconstructed with a validated technique. Four patients contributed bilateral knees without adjustment for clustering. Importantly, only four medial OA progression or ON events occurred, limiting definitive recommendations regarding postoperative alignment. Kaplan–Meier estimates at longer follow‐up should be interpreted cautiously due to declining numbers at risk. Finally, additional medial UKAs were classified as reoperations rather than lateral component revisions, despite representing clinically relevant secondary arthroplasty procedures.

## CONCLUSION

Fixed‐bearing lateral UKA in patients aged 55 years or younger demonstrated 100% lateral component revision‐free survival and favourable postoperative functional outcomes at a mean follow‐up of 5 years. No implant‐related failures such as aseptic loosening were observed. Seven knees underwent reoperation, including four additional medial UKAs for medial‐compartment OA progression or ON. Progression of OA in the medial compartment was the most common reason for reoperation. The observed relationship between postoperative near‐neutral or varus alignment and medial‐compartment OA progression or ON is limited by the small number of events and requires further investigation.

## AUTHOR CONTRIBUTIONS

**Solongo Abdulai**: Conceptualisation; data curation; formal analysis; investigation; methodology; visualisation; writing—original draft. **Conradin Schweizer**: Conceptualisation; data curation; writing—original draft. **Valentin Rühle**: Conceptualisation; data curation; writing—review and editing. **Tatjana Krug**: Conceptualisation; methodology; project administration; software; writing—review and editing. **Raffaela Morgenbesser**: Conceptualisation; writing—review and editing. **Joachim Herre**: Conceptualisation; writing—review and editing. **Peter R. Aldinger**: Conceptualisation; writing—review and editing. **Christian Merle**: Conceptualisation; methodology; project administration; supervision; validation; writing—review and editing. **Wenzel Waldstein**: Conceptualisation; methodology; project administration; supervision; validation; writing—review and editing.

## FUNDING INFORMATION

The authors have no funding to report.

## CONFLICT OF INTEREST STATEMENT

Peter R. Aldinger, Joachim Herre and Christian Merle report paid consultancy relationships with Zimmer Biomet and Medacta. Christian Merle and Christian Merle also receive research support from Zimmer Biomet and Aesculap. All remaining authors declare that they have no competing interests.

## ETHICS STATEMENT

Ethical approval for this study was granted by the Institutional Review Board (IRB_003/2023). The study was conducted in accordance with the ethical standards of the 1964 Declaration of Helsinki. All individual participants included in the study provided informed consent.

## Data Availability

Data supporting the results of this study may be obtained from the corresponding author upon reasonable request.
